# Comprehensive Genomic Characterization of A Case of Granular Cell Tumor of the Posterior Pituitary Gland: A Case Report

**DOI:** 10.3389/fendo.2021.762095

**Published:** 2021-12-01

**Authors:** Christopher S. Hong, Aladine A. Elsamadicy, Adeniyi Fisayo, Silvio E. Inzucchi, Pallavi P. Gopal, Eugenia M. Vining, E. Zeynep Erson-Omay, Sacit Bulent Omay

**Affiliations:** ^1^ Department of Neurosurgery, Yale School of Medicine, New Haven, CT, United States; ^2^ Department of Ophthalmology and Visual Science, Yale School of Medicine, New Haven, CT, United States; ^3^ Section of Endocrinology, Department of Medicine, Yale School of Medicine, New Haven, CT, United States; ^4^ Department of Pathology, Yale School of Medicine, New Haven, CT, United States; ^5^ Division of Otolaryngology, Department of Surgery, Yale School of Medicine, New Haven, CT, United States

**Keywords:** pituitary, granular cell, sequencing, SETD2, PAX8, case report

## Abstract

Granular cell tumors of the pituitary belong to a rare family of neoplasms, arising from the posterior pituitary gland. Although considered benign, they may cause significant morbidity and residual disease after resection can lead to poor clinical outcomes. Currently, there is no known medical therapy for any posterior pituitary gland tumor, in part due to sparse molecular characterization of these lesions. We report data from whole exome sequencing of a case of granular cell tumor of the pituitary, performed under an institutional review board approved protocol. A 77 year-old female underwent resection of an incidentally diagnosed pituitary mass that was causing radiographic compression of the optic nerves with a subclinical temporal field defect and central hypothyroidism. The pathology of the resected specimen demonstrated a granular cell tumor of the posterior pituitary gland. Whole-exome sequencing revealed mutations predicted to be deleterious in key oncogenes, *SETD2* and *PAX8*, both of which have been described in other cancers and could potentially be amenable to targeted therapies with existing approved drugs, including immune checkpoint inhibitors and histone deacetylase inhibitors, respectively. To our knowledge, this is the first comprehensive genomic characterization of granular cell tumor of the posterior pituitary gland. We report mutations in oncogenes predicted to be deleterious and reported in other cancers with potential for therapeutic targeting with existing pharmacologic agents. These data provide new insights into the molecular pathogenesis of GCT of the pituitary and may warrant further investigation.

## Introduction

Granular cell tumors (GCT) of the pituitary are rare, benign WHO I tumors. They, alongside pituicytomas, spindle cell oncocytomas, and sellar ependymomas comprise a family of tumors of the posterior pituitary gland (1). According to the latest edition of the WHO Classification of CNS Tumors, these pathologies may represent morphological variants of the same tumor with shared nuclear thyroid transcription factor-1 (TTF-1) expression and a lack of expression of pituitary hormones (2). Clinically, these tumors may cause symptoms related to local mass effect, including optic chiasm compression leading to visual field deficits, stalk compression with resultant hyperprolactinemia, and partial or even panhypopituitarism. Like pituitary adenomas, surgical resection of GCTs and other posterior pituitary tumors remains the mainstay of treatment (3). While considered benign, GCTs can be quite vascular (4–6), making complete surgical resection more difficult, and may lead to higher rates of tumor recurrence after subtotal resection and poorer survival outcomes (7, 8). The success of radiation therapy for residual tumors remains unclear, and likewise, there remain no known medical therapies for surgically refractory cases (7, 9, 10). As such, a better molecular understanding of these tumors is needed in order to develop potential novel targeted therapies.

To date, the molecular characterization of posterior pituitary tumors remain sparse and genomic sequencing have been comprised of only limited targeted sequencing panels (2, 11). To date, no genetic studies of GCTs of the pituitary have been performed. In this study, we report results from whole exome sequencing of a case of a GCT and discuss the relevant findings.

## Materials and Methods

This study was conducted under an institutional review board approved protocol at Yale University. The patient’s blood and tumor tissue were collected after obtaining written informed consent. Histopathology, including immunohistochemical studies, were evaluated by a board-certified neuropathologist.

Whole exome sequencing and analysis was performed in accordance with our previously described methods at the Yale Cancer for Genome Analysis (YCGA) (12). Briefly, genomic DNA from the tumor and blood were isolated and coding regions were captured with IDT xGen Exome Research Panel v1 (Integrated DNA Technologies, Coralville, IA) with the additional spike-in of ~2,500 regions totaling ~620 kb of RefGene coding regions together with custom spikes designed specifically for cancer by Genomic Oncology Academic Laboratory (GOAL) Consortium. The captured regions were then sequenced on the Illumina NovaSeq6000 whole-exome sequencing platform with 2x100 base pair reads. Downstream analysis of raw reads, including alignment, duplicate marking, realignment, and base quality recalibration was performed according to “GATK Best Practice” recommendations (GATK v4.1.9). Somatic single nucleotide variant (SNV), insertion/deletions (INDEL) and, and copy number variations (CNV) were identified as previously described (12). Mean coverage of 130.1x was achieved for blood and 265.8x for tumor tissue.

## Results

### Case Presentation

**Figure 1:**
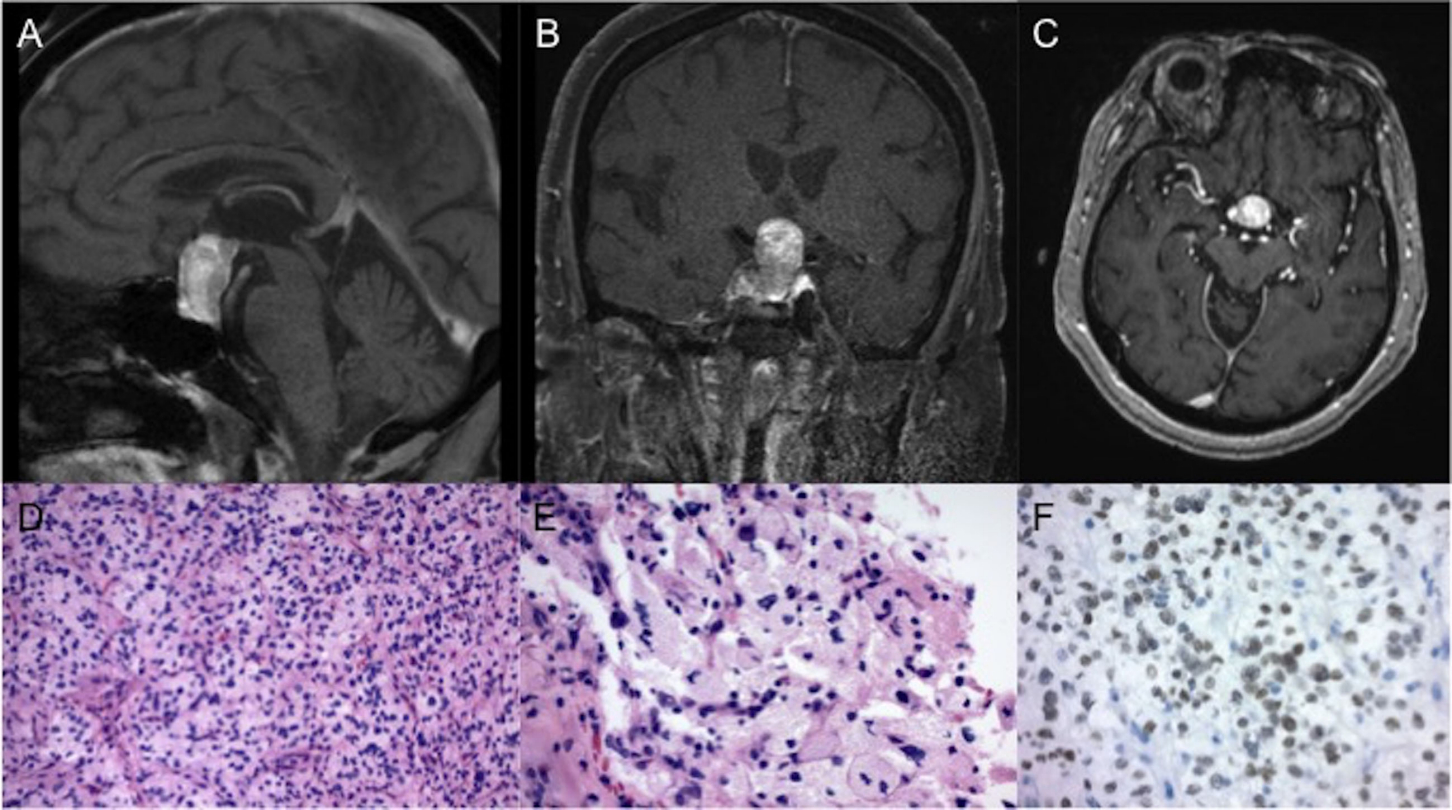
Imaging and pathology of the tumor. Representative **(A)** sagittal **(B)** coronal and **(C)** axial slices of a T1-weighted post-contrast MRI, obtained prior to surgery, showed a pituitary mass, measuring 1.6x1.5x2.6 cm. Final pathology of the resected tumor revealed a low grade neoplasm of polygonal cells with mild nuclear pleomorphism and granular cytoplasm on routine hematoxylin and eosin staining at **(D)** 200x and **(E)** 400x magnification. **(F)** Immunohistochemical stains revealed strong positivity for TTF-1 (magnification 400x).

A 77 year-old female with a recent diagnosis of ductal carcinoma of the breast in-situ with subsequent radiation and no radiographic evidence of recurrence was incidentally found to have a pituitary mass, measuring 1.6x1.5x2.6 cm on magnetic resonance imaging (MRI) of the brain, performed for a work-up of worsening hearing loss (**Figures 1A–C**). Review of systems was unremarkable and she was neurologically intact without any other complaints. Endocrine evaluation was notable for a mildly elevated prolactin of 51.1 ng/ml (normal range: 4.8-23.3 ng/mL), felt to be due to stalk effect, and decreased free T4 of 0.63 ng/dL (normal range: 0.80-1.70 ng/dL), the latter suggesting central hypothyroidism. She also demonstrated inappropriately low levels of LH and FSH gonadotropins for her menopausal status but of no clinical significance. The extent of pre-operative endocrine testing is outlined in **Table 1**. Neuro-ophthalmology testing revealed a temporal defect in the right eye, while left eye testing was limited given congenital vision loss on this side due to a macular scar. Given her visual deficit and radiographic compression of the optic nerves on MRI, surgical resection was recommended. The tumor was removed *via* an endoscopic endonasal approach and was noted intra-operatively to be well-encapsulated and originating from the posterior pituitary. The patient’s post-operative course was only remarkable for continued mildly reduced free T4 of 0.47 ng/dL and for a new low-normal morning cortisol level of 6.3 ug/dL (normal range: 6.0-18.4 ug/dL) for which she remained asymptomatic. She was started on levothyroxine and a prednisone taper, respectively. She was monitored for diabetes insipidus with serial sodium levels and urine output measurements but did not develop the condition. She also reported subjectively improved vision.

**Table 1:** Pre-operative endocrine testing of index patient.

Hormone	Value	Normal range
LH	<1.0	7.7 - 58.5 mIU/mL
FSH	2.2	25.8 - 134.8 mIU/mL
IGF-1	44	34 - 245 ng/mL
Cortisol	7.7	6.0 - 18.4 ug/dL
Prolactin	51.1	4.8 - 23.3 ng/mL
TSH	2.91	0.270-4.200 µIU/mL
Free T4	0.63	0.80-1.70 ng/dL

Final pathology revealed a low grade neoplasm composed of polygonal cells with discrete cell borders, mild nuclear pleomorphism, and ample granular to foamy cytoplasm (**Figures 1D, E**). PAS and PAS with diastase stains showed scattered tumor cells with diastase resistant granules. Immunohistochemical stains revealed strong positivity for S100 and TTF-1 (**Figure 1F**) with focal positivity for CD68. Tumor cells were negative for GFAP, synaptophysin, keratins (Cam5.2, pancytokeratin), inhibin, carbonic anhydrase, PAX8, and D2-40. The Ki-67 index was low at 1-2%. Taken together, given positive TTF-1 staining in the absence of cytokeratin expression, the immunoprofile of the surgical specimen was consistent with a diagnosis of GCT of the pituitary.

### Genomic Analysis

Whole-exome sequencing (WES) was performed on the resected surgical specimen and matched blood in accordance with an institutional review board-approved protocol and utilizing previously described methods (13). No germline variants of potential pathological significance were identified. The analysis of somatic SNV/INDEL data revealed two somatic missense mutations in *SETD2* (c.T2081C:p.V694A) and *PAX8* (c.T527A:p.L176Q) with variant allele frequency (VAF) of 22.1% and 25.5%, respectively. Both variants are considered pathogenic by FATHMM algorithm (14). Detailed data are provided in **Table 2**. Chromosomal analysis revealed deletions in chromosomes 9, 10, and 13. Additionally, there was focal amplification on chromosome 12, involving *PIK3C2G*, a member of the class II PI3K kinase family, whose dysregulation may be involved in human metabolic diseases but its role in cancer remains unclear (15).

**Table 2:** Somatic genetic findings of index patient.

Gene	Chromosome	Position	Ref	Alt	HGVS (RefGene)	dbSNP
SETD2	3	47164045	A	G	NM_014159:exon3:c.T2081C:p.V694A	rs786201856
PAX8	2	47164045	A	T	NM_003466:exon6:c.T527A:p.L176Q	rs587779780

## Discussion

Outside of the posterior pituitary gland, GCTs have been described in many anatomic locations, most commonly in skin/subcutaneous soft tissues and the gastrointestinal tract and are thought to arise from Schwann cell origin (16). All GCTs stain positive for S100 and histologically show cells with granular eosinophilic cytoplasm, as demonstrated in our case. WES of GCTs remain limited, but recent hallmark studies have demonstrated loss-of-function mutations in vacuolar ATPase subunits, leading to aberrant lysosomal pH regulation and accumulation of intracytoplasmic granules, as observed histologically in GCTs (17–19). While these ATPase mutations may occur in up to 72% of GCTs (18), it is not known if they represent true driver mutations in tumorigenesis. We did not observe genomic alterations in ATPases or other genes integral to endosomal/lysosomal networks in our patient (17). It remains unclear whether GCTs of the posterior pituitary are true GCTs of similar Schwann cell origin. Alternatively, they have been proposed to belong to a morphologic spectrum of posterior pituitary tumors, all derived from pituicyte origin (20). Further genomic characterization of GCTs of the posterior pituitary may help elucidate this distinction.

Genomic characterization of posterior pituitary tumors have been limited to a handful of cases, given the rarity of these pathologies and limited sample sizes taken at time of surgery (11). For spindle cell oncocytomas and pituicytomas, individual mutations affecting the MAPK signaling pathway (*SND1, FAT1, HRAS*), as well as in other oncogenes (i.e. *MEN1, TSC1, CBL, FZD7, PIK3GC, SBK1, CDKN2A/B, SKT11, SMARCA4, CIC, SMARCB1, NF1, NF2*) have been reported in case reports or limited case series (11, 21, 22). Interestingly, *BRAF* mutations have been identified in two cases of spindle cell oncocytomas, which responded to targeted therapy with BRAF inhibitors (23, 24). However, to date, a comprehensive genetic study of GCTs of the pituitary has not been reported in the literature.

In this study, we found somatic mutations in *SETD2* and *PAX8*, which are known tumor-related genes but not reported in tumors of the posterior pituitary. *SETD2* is a histone methyltransferase whose normal function is critical for genomic stability and DNA damage repair (25). Clinically, *SETD2* mutations are most prevalent in clear cell renal carcinomas but are also less commonly seen in hematopoietic cancers, high-grade glioma, melanoma, and adenocarcinomas of the lung and gastrointestinal system (25). *SETD2* mutations have been correlated with shorter progression-free and overall survivals in metastatic renal cell carcinoma and breast cancers (26). Interestingly, SETD2 depletion *in vitro* led to microsatellite instability and increased mutational burden, characteristic of the mismatch repair deficient phenotype that may be amenable to immunotherapies (27). Indeed, in an analysis of The Cancer Genome Atlas (TCGA) pan-cancer cohort found that patients with tumors harboring *SETD2* mutations responded favorably to treatment with immune checkpoint inhibitors, raising the possibility for targeted therapies in *SETD2*-mutated cancers (28).

PAX8 is a member of the paired box family of transcription factors, which encode proteins important in embryogenesis, particularly for thyroid and urogenital system development. In cancer, mutations in *PAX8* have multiple downstream consequences affecting cell proliferation, adhesion, and angiogenesis (29). Clinically, positive immunohistochemical staining for PAX8 is widely used to diagnose primary renal cell tumors and mutations are also frequently seen in thyroid, ovarian, bladder, prostate, endometrial, cervical, and uterine cancers (29). In our case, the tumor did not stain for PAX8, as well as carbonic anhydrase and keratins, effectively ruling out a diagnosis of renal cell carcinoma. Although cases of pituitary metastasis have been reported from *PAX8*-mutated renal cell carcinoma (30), *PAX8* mutations have not been reported in primary pituitary pathologies. In addition, PAX8 is a promising therapeutic target as its expression is restricted in normal tissues, compared to its role in cancer (31). Several preclinical studies have demonstrated suppression of cancer growth with genetic knockdown or pharmacologic inhibition of PAX8 (32, 33), including histone deacetylases (HDAC) inhibitors, which may epigenetically downregulate PAX8 transcripts and are a class of small molecule drugs now approved for cancer therapy (34).

This study reports comprehensive genomic characterization of a case of GCT of the pituitary. We found mutations predicted to be deleterious in known oncogenes, *SETD2* and *PAX8*, previously not described in this pathology but have potential for therapeutic targeting with existing pharmacologic agents. The significance of these findings is limited by being a single case report, but genomic characterization of this rare tumor entity remains severely lacking. Further gene sequencing studies and mechanistic preclinical data are needed to better understand the pathophysiology and potential for molecularly based targeted therapies for GCTs of the pituitary.

## Data Availability Statement

The data used to support the findings of this study are available from the corresponding author upon request.

## Ethics Statement

The studies involving human participants were reviewed and approved by Yale University Institutional Review Board. The patients/participants provided their written informed consent to participate in this study.

## Author Contributions

CH, ZE-O, and SB designed the study. All authors provided medical care to the patient. CH and ZE-O performed the genetic analysis. CH, ZE-O, and SB wrote the manuscript. All authors contributed to the article and approved the submitted version.

## Funding

Funding for performing genomic sequencing and analysis of the data was provided by the Yale School of Medicine, Department of Neurosurgery Clinical Sequencing Funds.

## Conflict of Interest

The authors declare that the research was conducted in the absence of any commercial or financial relationships that could be construed as a potential conflict of interest.

## Publisher’s Note

All claims expressed in this article are solely those of the authors and do not necessarily represent those of their affiliated organizations, or those of the publisher, the editors and the reviewers. Any product that may be evaluated in this article, or claim that may be made by its manufacturer, is not guaranteed or endorsed by the publisher.
